# Bis(*S*-benzyl­isothio­uronium) tetra­chloridozincate(II)

**DOI:** 10.1107/S1600536808015195

**Published:** 2008-05-30

**Authors:** D. Gayathri, D. Velmurugan, P. Hemalatha, V. Veeravazhuthi, K. Ravikumar

**Affiliations:** a Centre of Advanced Study in Crystallography and Biophysics, University of Madras, Guindy Campus, Chennai 600 025, India; b Department of Physics, PSG College of Technology, Coimbatore 641 004, India; c Department of Physics, PSG College of Arts and Science, Coimbatore 641 101, India; d Laboratory of X-ray Crystallography, Indian Institute of Chemical Technology, Hyderabad 500 007, India

## Abstract

The asymmetric unit of the title compound, (C_8_H_11_N_2_S)_2_[ZnCl_4_], contains two *S*-benzyl­isothio­uronium cations which differ in the C—C—S—C torsion angle [165.3 (2) and 81.9 (2)°] and a tetrahedral tetra­chloridozincate anion. The crystal structure is stabilized by N—H⋯Cl, C—H⋯Cl and C—H⋯S inter­actions.

## Related literature

For related literature, see: Hemalatha *et al.* (2006 ▶); Zhang *et al.* (1994 ▶); Barker & Powell (1998 ▶).
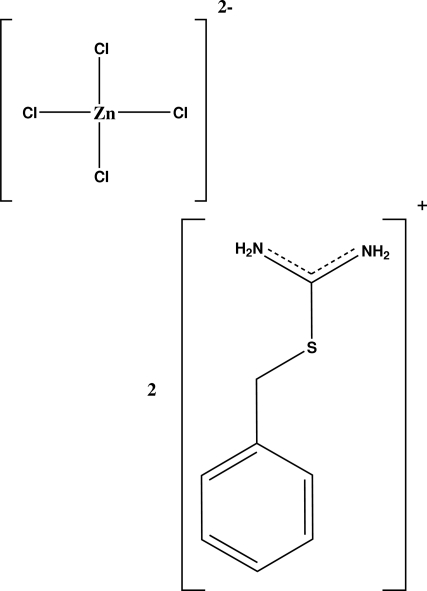


## Experimental

### 

#### Crystal data

(C_8_H_11_N_2_S)_2_[ZnCl_4_]
*M* *_r_* = 541.67
Monoclinic, 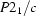

*a* = 15.2135 (11) Å
*b* = 6.4475 (5) Å
*c* = 23.9277 (18) Å
β = 95.368 (1)°
*V* = 2336.8 (3) Å^3^
*Z* = 4
Mo *K*α radiation
μ = 1.70 mm^−1^
*T* = 293 (2) K
0.27 × 0.23 × 0.21 mm

#### Data collection

Bruker SMART APEX diffractometer
Absorption correction: none
25129 measured reflections
5481 independent reflections
4986 reflections with *I* > 2σ(*I*)
*R*_int_ = 0.026

#### Refinement

*R*[*F* ^2^ > 2σ(*F* ^2^)] = 0.029
*wR*(*F* ^2^) = 0.073
*S* = 1.08
5481 reflections
244 parameters
H-atom parameters constrained
Δρ_max_ = 0.41 e Å^−3^
Δρ_min_ = −0.32 e Å^−3^

### 

Data collection: *SMART* (Bruker, 2001 ▶); cell refinement: *SAINT* (Bruker, 2001 ▶); data reduction: *SAINT*; program(s) used to solve structure: *SHELXS97* (Sheldrick, 2008 ▶); program(s) used to refine structure: *SHELXL97* (Sheldrick, 2008 ▶); molecular graphics: *PLATON* (Spek, 2003 ▶); software used to prepare material for publication: *SHELXL97* and *PARST* (Nardelli, 1995 ▶).

## Supplementary Material

Crystal structure: contains datablocks I, global. DOI: 10.1107/S1600536808015195/bt2708sup1.cif

Structure factors: contains datablocks I. DOI: 10.1107/S1600536808015195/bt2708Isup2.hkl

Additional supplementary materials:  crystallographic information; 3D view; checkCIF report

## Figures and Tables

**Table 1:** Selected bond lengths (Å)

Cl1—Zn1	2.2792 (5)
Cl2—Zn1	2.2650 (5)
Cl3—Zn1	2.2718 (5)
Cl4—Zn1	2.2589 (5)

**Table 2:** Hydrogen-bond geometry (Å, °)

*D*—H⋯*A*	*D*—H	H⋯*A*	*D*⋯*A*	*D*—H⋯*A*
N1—H1*A*⋯Cl3	0.86	2.68	3.372 (2)	139
N1—H1*B*⋯Cl2^i^	0.86	2.45	3.255 (2)	157
N2—H2*A*⋯Cl4	0.86	2.53	3.219 (2)	138
N2—H2*B*⋯Cl3^ii^	0.86	2.62	3.262 (2)	132
N3—H3*A*⋯Cl1^iii^	0.86	2.72	3.469 (2)	147
N3—H3*A*⋯Cl4^iii^	0.86	2.65	3.244 (2)	128
N3—H3*B*⋯Cl1^iv^	0.86	2.44	3.283 (2)	166
N4—H4*A*⋯Cl1^iii^	0.86	2.49	3.290 (2)	156
N4—H4*B*⋯Cl2^i^	0.86	2.62	3.447 (2)	163
C15—H15*A*⋯S1	0.97	2.87	3.591 (2)	132
C15—H15*A*⋯Cl2^v^	0.97	2.77	3.556 (2)	139
C15—H15*B*⋯Cl2^i^	0.97	2.65	3.594 (2)	164

Symmetry codes: (i) 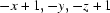; (ii) 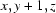; (iii) 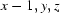; (iv) 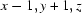; (v) 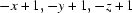.

**Fig. 1.**
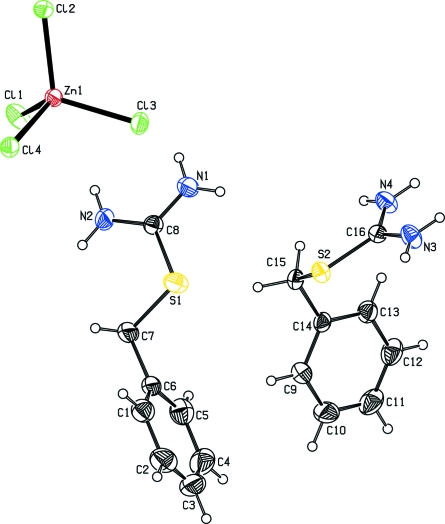
The molecular structure of title compound, showing 30% probability displacement ellipsoids.

**Fig. 2.**
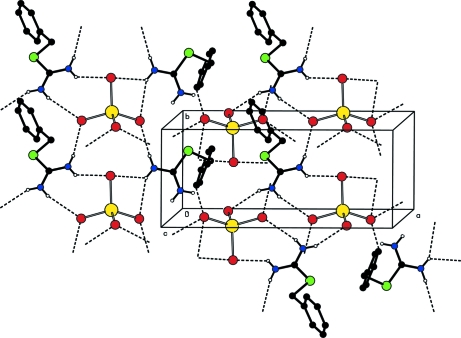
The molecular packing of the title compound showing N—H···Cl interactions viewed down c axis.

**Fig. 3.**
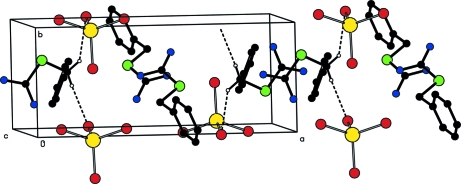
The molecular packing of the title compound showing C—H···Cl interactions viewed down c axis.

## supplementary crystallographic information

### Comment

Most organic nonlinear optical (NLO) crystals have usually poor mechanical and thermal properties, and are susceptible to damage during processing. It is difficult to grow large optical quality crystals of these materials for device applications (Zhang *et al.*,  1994). For further enhancement of NLO property many efforts have been made on developing new semiorganic NLO materials. The title compound is one of the new metalorganic nonlinear optical crystals. It combines the advantages of both organic and inorganic materials.

The C—N, S—C bond lengths and C—S—C and N—C—N bond angles are comparable with the similar structure reported earlier (Barker & Powell, 1998). There is a difference in the torsion angles C6—C7—S1—C8 [165.3 (2)°] and C14—C15—S2—C16 [81.9 (2)°] in the two molecules which indicates a difference in the conformation of the two molecules in the asymmetric unit.

The crystal structure (Figs. 2 and 3) is stabilized by N—H···Cl, C—H···Cl and C—H···S  interactions.

### Experimental

First *S*-benzylisothiouronium chloride (SBTC) was synthesized as discussed in an earlier report (Hemalatha *et al.*, 2006). The solutions of SBTC (5 g m) and zinc chloride (1 g m) were prepared separately in minimum amount of water. Then the solutions were mixed together and stirred for 1 hr at 45°C. The resulting complex was filtered and thoroughly washed with distilled water. The product was recrystallized repeatedly from 0.2 *M* hydrochloric solution to grow transparent and good quality single crystals for NLO applications. Needle shape crystals were obtained from the saturated solution (with water) of the title compound by slow evaporation technique at room temperature.

### Refinement

All H-atoms were refined using a riding model with d(C—H) = 0.93 Å or d(N—H) = 0.86 Å *U*_iso_=1.2*U*_eq_ (C,N)  and 0.97 Å, *U*_iso_ = 1.2*U*_eq_ (C) for CH_2_.

### Crystal data

**Table:**

(C_8_H_11_N_2_S)_2_[ZnCl_4_]	*F*_000_ = 1104
*M**_r_* = 541.67	*D*_x_ = 1.540 Mg m^−^^3^
Monoclinic, *P*2_1_/*c*	Mo *K*α radiation λ = 0.71073 Å
Hall symbol: -P 2ybc	Cell parameters from 2794 reflections
*a* = 15.2135 (11) Å	θ = 1.3–25.0º
*b* = 6.4475 (5) Å	µ = 1.70 mm^−^^1^
*c* = 23.9277 (18) Å	*T* = 293 (2) K
β = 95.3680 (10)º	Needle, colorless
*V* = 2336.8 (3) Å^3^	0.27 × 0.23 × 0.21 mm
*Z* = 4	

### Data collection

**Table:**

Bruker SMART APEX diffractometer	4986 reflections with *I* > 2σ(*I*)
Radiation source: fine-focus sealed tube	*R*_int_ = 0.026
Monochromator: graphite	θ_max_ = 28.0º
*T* = 293(2) K	θ_min_ = 1.3º
ω scans	*h* = −20→20
Absorption correction: none	*k* = −8→8
25129 measured reflections	*l* = −30→31
5481 independent reflections	

### Refinement

**Table:**

Refinement on *F*^2^	Secondary atom site location: difference Fourier map
Least-squares matrix: full	Hydrogen site location: inferred from neighbouring sites
*R*[*F*^2^ > 2σ(*F*^2^)] = 0.029	H-atom parameters constrained
*wR*(*F*^2^) = 0.073	*w* = 1/[σ^2^(*F*_o_^2^) + (0.0325*P*)^2^ + 1.0433*P*] where *P* = (*F*_o_^2^ + 2*F*_c_^2^)/3
*S* = 1.08	(Δ/σ)_max_ = 0.001
5481 reflections	Δρ_max_ = 0.41 e Å^−^^3^
244 parameters	Δρ_min_ = −0.32 e Å^−^^3^
Primary atom site location: structure-invariant direct methods	Extinction correction: none

### Special details

**Table:**

Geometry. All e.s.d.'s (except the e.s.d. in the dihedral angle between two l.s. planes) are estimated using the full covariance matrix. The cell e.s.d.'s are taken into account individually in the estimation of e.s.d.'s in distances, angles and torsion angles; correlations between e.s.d.'s in cell parameters are only used when they are defined by crystal symmetry. An approximate (isotropic) treatment of cell e.s.d.'s is used for estimating e.s.d.'s involving l.s. planes.
Refinement. Refinement of *F*^2^ against ALL reflections. The weighted *R*-factor *wR* and goodness of fit *S* are based on *F*^2^, conventional *R*-factors *R* are based on *F*, with *F* set to zero for negative *F*^2^. The threshold expression of *F*^2^ > σ(*F*^2^) is used only for calculating *R*-factors(gt) *etc*. and is not relevant to the choice of reflections for refinement. *R*-factors based on *F*^2^ are statistically about twice as large as those based on *F*, and *R*- factors based on ALL data will be even larger.

### Fractional atomic coordinates and isotropic or equivalent isotropic displacement parameters (Å^2^)

**Table:**

	*x*	*y*	*z*	*U*_iso_*/*U*_eq_	
S1	0.38843 (3)	0.61059 (12)	0.36896 (3)	0.06677 (19)	
N1	0.42614 (13)	0.2947 (3)	0.43326 (8)	0.0562 (5)	
H1A	0.4589	0.2133	0.4547	0.067*	
H1B	0.3709	0.2676	0.4258	0.067*	
C1	0.36865 (17)	1.0458 (4)	0.28381 (11)	0.0598 (6)	
H1	0.3774	1.1179	0.3175	0.072*	
C2	0.31899 (18)	1.1338 (5)	0.23884 (15)	0.0783 (8)	
H2	0.2949	1.2652	0.2423	0.094*	
C3	0.3052 (2)	1.0311 (7)	0.19011 (14)	0.0869 (10)	
H3	0.2718	1.0920	0.1600	0.104*	
C4	0.3395 (2)	0.8398 (7)	0.18442 (11)	0.0867 (10)	
H4	0.3293	0.7693	0.1505	0.104*	
C5	0.39016 (18)	0.7482 (4)	0.22921 (12)	0.0663 (7)	
H5	0.4137	0.6165	0.2252	0.080*	
C6	0.40541 (13)	0.8523 (4)	0.27928 (9)	0.0475 (5)	
C7	0.46069 (15)	0.7581 (4)	0.32779 (11)	0.0664 (7)	
H7A	0.4903	0.8661	0.3506	0.080*	
H7B	0.5052	0.6680	0.3143	0.080*	
C8	0.46009 (12)	0.4585 (3)	0.41203 (8)	0.0399 (4)	
S2	0.06186 (3)	0.66804 (7)	0.41032 (2)	0.04038 (11)	
N2	0.54328 (12)	0.5038 (3)	0.42243 (9)	0.0555 (5)	
H2A	0.5772	0.4246	0.4438	0.067*	
H2B	0.5644	0.6130	0.4079	0.067*	
C9	0.15114 (15)	0.6634 (4)	0.27656 (10)	0.0578 (6)	
H9	0.1742	0.7917	0.2880	0.069*	
C10	0.13285 (18)	0.6205 (6)	0.22003 (11)	0.0757 (8)	
H10	0.1442	0.7203	0.1936	0.091*	
C11	0.09845 (18)	0.4338 (5)	0.20262 (11)	0.0743 (8)	
H11	0.0859	0.4075	0.1645	0.089*	
C12	0.08244 (17)	0.2855 (5)	0.24096 (10)	0.0651 (7)	
H12	0.0588	0.1583	0.2290	0.078*	
C13	0.10135 (14)	0.3240 (4)	0.29755 (9)	0.0505 (5)	
H13	0.0914	0.2213	0.3235	0.061*	
C14	0.13496 (11)	0.5144 (3)	0.31602 (8)	0.0395 (4)	
C15	0.15495 (11)	0.5601 (3)	0.37715 (8)	0.0377 (4)	
H15A	0.2038	0.6572	0.3818	0.045*	
H15B	0.1737	0.4330	0.3964	0.045*	
C16	−0.00143 (12)	0.4549 (3)	0.42524 (8)	0.0373 (4)	
N3	−0.08381 (12)	0.4941 (3)	0.43308 (8)	0.0527 (4)	
H3A	−0.1180	0.3953	0.4418	0.063*	
H3B	−0.1038	0.6187	0.4295	0.063*	
N4	0.03000 (12)	0.2657 (3)	0.43054 (8)	0.0519 (4)	
H4A	−0.0036	0.1658	0.4392	0.062*	
H4B	0.0842	0.2415	0.4253	0.062*	
Cl1	0.85432 (4)	−0.02098 (8)	0.44408 (3)	0.05782 (15)	
Cl2	0.76342 (3)	−0.05687 (7)	0.57924 (2)	0.04173 (11)	
Cl3	0.60408 (3)	−0.01885 (8)	0.44970 (3)	0.05317 (14)	
Cl4	0.73327 (3)	0.42677 (7)	0.49034 (2)	0.04099 (11)	
Zn1	0.738101 (13)	0.07662 (3)	0.491654 (9)	0.03552 (7)	

### Atomic displacement parameters (Å^2^)

**Table:**

	*U*^11^	*U*^22^	*U*^33^	*U*^12^	*U*^13^	*U*^23^
S1	0.0303 (2)	0.0860 (4)	0.0846 (4)	0.0098 (3)	0.0085 (2)	0.0446 (4)
N1	0.0452 (10)	0.0590 (11)	0.0640 (12)	−0.0027 (8)	0.0029 (8)	0.0212 (9)
C1	0.0585 (14)	0.0609 (14)	0.0607 (14)	−0.0017 (11)	0.0088 (11)	0.0033 (11)
C2	0.0582 (15)	0.0761 (18)	0.101 (2)	0.0120 (14)	0.0101 (15)	0.0311 (18)
C3	0.0568 (16)	0.128 (3)	0.073 (2)	−0.0069 (18)	−0.0079 (14)	0.039 (2)
C4	0.079 (2)	0.135 (3)	0.0459 (14)	−0.027 (2)	0.0053 (13)	−0.0046 (17)
C5	0.0617 (15)	0.0708 (16)	0.0684 (16)	−0.0033 (12)	0.0167 (12)	−0.0063 (13)
C6	0.0359 (9)	0.0567 (12)	0.0498 (11)	−0.0044 (9)	0.0043 (8)	0.0124 (10)
C7	0.0423 (12)	0.0819 (18)	0.0735 (16)	−0.0119 (11)	−0.0031 (11)	0.0342 (13)
C8	0.0349 (9)	0.0424 (10)	0.0426 (10)	0.0044 (7)	0.0041 (7)	0.0031 (8)
S2	0.0369 (2)	0.0350 (2)	0.0500 (3)	−0.00065 (18)	0.00816 (19)	−0.00128 (19)
N2	0.0386 (9)	0.0523 (10)	0.0724 (13)	−0.0025 (8)	−0.0125 (8)	0.0143 (9)
C9	0.0515 (12)	0.0654 (14)	0.0565 (13)	−0.0130 (11)	0.0048 (10)	0.0116 (11)
C10	0.0635 (15)	0.113 (2)	0.0521 (14)	−0.0118 (16)	0.0110 (12)	0.0252 (15)
C11	0.0585 (15)	0.123 (3)	0.0408 (12)	−0.0074 (16)	0.0039 (11)	−0.0090 (14)
C12	0.0599 (14)	0.0816 (17)	0.0530 (13)	−0.0095 (13)	0.0019 (11)	−0.0212 (12)
C13	0.0502 (12)	0.0544 (12)	0.0466 (11)	−0.0072 (10)	0.0025 (9)	−0.0054 (9)
C14	0.0277 (8)	0.0497 (10)	0.0409 (10)	−0.0010 (7)	0.0021 (7)	−0.0001 (8)
C15	0.0285 (8)	0.0400 (9)	0.0442 (10)	−0.0021 (7)	0.0004 (7)	−0.0029 (7)
C16	0.0372 (9)	0.0397 (9)	0.0351 (9)	−0.0016 (7)	0.0040 (7)	0.0021 (7)
N3	0.0406 (9)	0.0461 (9)	0.0741 (12)	−0.0004 (7)	0.0188 (8)	0.0060 (9)
N4	0.0483 (10)	0.0402 (9)	0.0687 (12)	0.0002 (7)	0.0130 (9)	0.0119 (8)
Cl1	0.0586 (3)	0.0431 (3)	0.0774 (4)	0.0024 (2)	0.0365 (3)	−0.0026 (2)
Cl2	0.0459 (2)	0.0367 (2)	0.0417 (2)	−0.00012 (18)	−0.00090 (18)	0.00612 (17)
Cl3	0.0417 (3)	0.0453 (3)	0.0688 (3)	−0.0069 (2)	−0.0143 (2)	0.0068 (2)
Cl4	0.0366 (2)	0.0346 (2)	0.0508 (3)	0.00228 (16)	−0.00096 (19)	0.00113 (18)
Zn1	0.03067 (11)	0.03633 (12)	0.03951 (12)	0.00107 (8)	0.00309 (8)	0.00358 (8)

### Geometric parameters (Å, °)

**Table:**

S1—C8	1.732 (2)	C9—C10	1.383 (4)
S1—C7	1.813 (2)	C9—C14	1.385 (3)
N1—C8	1.300 (3)	C9—H9	0.9300
N1—H1A	0.8600	C10—C11	1.362 (4)
N1—H1B	0.8600	C10—H10	0.9300
C1—C6	1.376 (3)	C11—C12	1.363 (4)
C1—C2	1.378 (4)	C11—H11	0.9300
C1—H1	0.9300	C12—C13	1.380 (3)
C2—C3	1.340 (5)	C12—H12	0.9300
C2—H2	0.9300	C13—C14	1.386 (3)
C3—C4	1.351 (5)	C13—H13	0.9300
C3—H3	0.9300	C14—C15	1.495 (3)
C4—C5	1.391 (4)	C15—H15A	0.9700
C4—H4	0.9300	C15—H15B	0.9700
C5—C6	1.374 (3)	C16—N3	1.309 (2)
C5—H5	0.9300	C16—N4	1.312 (2)
C6—C7	1.497 (3)	N3—H3A	0.8600
C7—H7A	0.9700	N3—H3B	0.8600
C7—H7B	0.9700	N4—H4A	0.8600
C8—N2	1.300 (3)	N4—H4B	0.8600
S2—C16	1.734 (2)	Cl1—Zn1	2.2792 (5)
S2—C15	1.825 (2)	Cl2—Zn1	2.2650 (5)
N2—H2A	0.8600	Cl3—Zn1	2.2718 (5)
N2—H2B	0.8600	Cl4—Zn1	2.2589 (5)
			
C8—S1—C7	103.9 (1)	C14—C9—H9	120.1
C8—N1—H1A	120.0	C11—C10—C9	120.8 (3)
C8—N1—H1B	120.0	C11—C10—H10	119.6
H1A—N1—H1B	120.0	C9—C10—H10	119.6
C6—C1—C2	120.6 (3)	C10—C11—C12	120.1 (2)
C6—C1—H1	119.7	C10—C11—H11	119.9
C2—C1—H1	119.7	C12—C11—H11	119.9
C3—C2—C1	120.4 (3)	C11—C12—C13	120.0 (2)
C3—C2—H2	119.8	C11—C12—H12	120.0
C1—C2—H2	119.8	C13—C12—H12	120.0
C2—C3—C4	120.6 (3)	C12—C13—C14	120.6 (2)
C2—C3—H3	119.7	C12—C13—H13	119.7
C4—C3—H3	119.7	C14—C13—H13	119.7
C3—C4—C5	120.1 (3)	C9—C14—C13	118.7 (2)
C3—C4—H4	120.0	C9—C14—C15	119.82 (19)
C5—C4—H4	120.0	C13—C14—C15	121.44 (18)
C6—C5—C4	120.0 (3)	C14—C15—S2	113.93 (12)
C6—C5—H5	120.0	C14—C15—H15A	108.8
C4—C5—H5	120.0	S2—C15—H15A	108.8
C5—C6—C1	118.3 (2)	C14—C15—H15B	108.8
C5—C6—C7	121.0 (2)	S2—C15—H15B	108.8
C1—C6—C7	120.6 (2)	H15A—C15—H15B	107.7
C6—C7—S1	108.00 (15)	N3—C16—N4	120.7 (2)
C6—C7—H7A	110.1	N3—C16—S2	115.7 (2)
S1—C7—H7A	110.1	N4—C16—S2	123.5 (2)
C6—C7—H7B	110.1	C16—N3—H3A	120.0
S1—C7—H7B	110.1	C16—N3—H3B	120.0
H7A—C7—H7B	108.4	H3A—N3—H3B	120.0
N1—C8—N2	121.5 (2)	C16—N4—H4A	120.0
N1—C8—S1	116.2 (2)	C16—N4—H4B	120.0
N2—C8—S1	122.3 (2)	H4A—N4—H4B	120.0
C16—S2—C15	104.82 (9)	Cl4—Zn1—Cl2	113.28 (2)
C8—N2—H2A	120.0	Cl4—Zn1—Cl3	103.76 (2)
C8—N2—H2B	120.0	Cl2—Zn1—Cl3	111.95 (2)
H2A—N2—H2B	120.0	Cl4—Zn1—Cl1	107.14 (2)
C10—C9—C14	119.7 (2)	Cl2—Zn1—Cl1	106.53 (2)
C10—C9—H9	120.1	Cl3—Zn1—Cl1	114.24 (3)
			
C6—C1—C2—C3	0.5 (4)	C14—C9—C10—C11	0.5 (4)
C1—C2—C3—C4	0.2 (5)	C9—C10—C11—C12	−0.7 (5)
C2—C3—C4—C5	−0.5 (5)	C10—C11—C12—C13	−0.2 (4)
C3—C4—C5—C6	−0.1 (4)	C11—C12—C13—C14	1.3 (4)
C4—C5—C6—C1	0.8 (4)	C10—C9—C14—C13	0.6 (3)
C4—C5—C6—C7	−179.1 (2)	C10—C9—C14—C15	179.8 (2)
C2—C1—C6—C5	−1.0 (4)	C12—C13—C14—C9	−1.5 (3)
C2—C1—C6—C7	178.9 (2)	C12—C13—C14—C15	179.3 (2)
C5—C6—C7—S1	−89.8 (2)	C9—C14—C15—S2	91.8 (2)
C1—C6—C7—S1	90.3 (2)	C13—C14—C15—S2	−89.0 (2)
C8—S1—C7—C6	165.34 (19)	C16—S2—C15—C14	81.90 (15)
C7—S1—C8—N1	−159.52 (19)	C15—S2—C16—N3	−159.78 (16)
C7—S1—C8—N2	20.0 (2)	C15—S2—C16—N4	22.5 (2)

### Hydrogen-bond geometry (Å, °)

**Table:**

*D*—H···*A*	*D*—H	H···*A*	*D*···*A*	*D*—H···*A*
N1—H1A···Cl3	0.86	2.68	3.372 (2)	139
N1—H1B···Cl2^i^	0.86	2.45	3.255 (2)	157
N2—H2A···Cl4	0.86	2.53	3.219 (2)	138
N2—H2B···Cl3^ii^	0.86	2.62	3.262 (2)	132
N3—H3A···Cl1^iii^	0.86	2.72	3.469 (2)	147
N3—H3A···Cl4^iii^	0.86	2.65	3.244 (2)	128
N3—H3B···Cl1^iv^	0.86	2.44	3.283 (2)	166
N4—H4A···Cl1^iii^	0.86	2.49	3.290 (2)	156
N4—H4B···Cl2^i^	0.86	2.62	3.447 (2)	163
C15—H15A···S1	0.97	2.87	3.591 (2)	132
C15—H15A···Cl2^v^	0.97	2.77	3.556 (2)	139
C15—H15B···Cl2^i^	0.97	2.65	3.594 (2)	164

Symmetry codes:  (i) −*x*+1, −*y*, −*z*+1; (ii) *x*, *y*+1, *z*; (iii) *x*−1, *y*, *z*; (iv) *x*−1, *y*+1, *z*; (v) −*x*+1, −*y*+1, −*z*+1.
